# Separation anxiety disorder in pregnancy: The relationship between attachment styles, childhood trauma, and prenatal attachment

**DOI:** 10.3389/fpsyt.2026.1723586

**Published:** 2026-03-03

**Authors:** Nehir Mutlusoy Eraslan, Rümeysa Yeni Elbay

**Affiliations:** 1 Psychiatry Department, Goztepe Prof Dr Suleyman Yalcin City Hospital, Istanbul, Türkiye; 2 Psychiatry Department, Faculty of Medicine, Istanbul Medeniyet University, Istanbul, Türkiye

**Keywords:** attachment styles, childhood trauma, pregnancy, prenatal attachment, separation anxiety disorder

## Abstract

**Background and aim:**

Separation anxiety, rooted in attachment theory, involves distress when separated from attachment figures. This study aims to investigate the prevalence Adult Separation Anxiety Disorder (ASAD) among pregnant women and to explore its associations with attachment styles, childhood trauma, and prenatal attachment.

**Method:**

This cross-sectional study included 106 third-trimester pregnant women, who were classified into ASAD (n = 51) and non-ASAD (n = 55) groups based on the Structured Clinical Interview for Separation Anxiety Symptoms (SAD-SCI). Participants were also assessed using the Structured Clinical Interview for DSM-5 (SCID-5), along with a sociodemographic data form, the Adult Attachment Style Scale (AASS), the Childhood Trauma Questionnaire-33 (CTQ-33), and the Prenatal Attachment Inventory (PAI). Statistical analyses included chi-square tests, t-tests, correlation analyses, and regression models.

**Results:**

ASAD was identified in 48.2% of the participants. Anxious/ambivalent attachment, avoidant attachment styles and childhood trauma were significantly associated with ASAD severity (p<0,001). In multivariable logistic regression analysis, anxious/ambivalent attachment style was independently associated with ASAD diagnosis (OR = 1.81; 95% CI: 1.085–3.021). Supportive parental bonding was associated with lower ASAD severity, whereas overprotective parenting attitudes were associated with higher severity. No significant relationship was found between ASAD and prenatal attachment (p>0.05), but childhood sexual abuse negatively correlated with prenatal attachment (p=0.012). Positive paternal care (p=0.004) and overprotective parenting attitudes (p=0.050) were associated with stronger prenatal attachment.

**Conclusion:**

ASAD is common during late pregnancy and is closely associated with insecure attachment patterns and adverse childhood experiences, but not with prenatal attachment. These findings support the clinical value of routine ASAD screening during late pregnancy and targeted interventions for at-risk women. Longitudinal studies are needed to clarify the postpartum course of ASAD and its implications for mother–infant relationships and child development.

## Introduction

Attachment is an enduring emotional bond that shapes lifelong relationship patterns (1, 2). Bowlby suggested that early caregiver interactions form internal working models, which in turn influence attachment (3–5). Ainsworth classified attachment styles influencing emotional regulation and interpersonal relationships as secure attachment and insecure attachment, including anxious/ambivalent and avoidant attachment styles (6, 7), subsequently, Main and Solomon introduced the disorganized attachment pattern within attachment classifications (8).

Secure attachment promotes resilience, whereas insecure attachment, especially anxious/ambivalent, is linked to psychiatric disorders such as PTSD, OCD, and depression (9–11). Disorganized attachment has been particularly associated with dissociative disorders (12). Research associates insecure attachment with separation anxiety disorder (SAD) (13–15). Among insecure attachment patterns, separation anxiety disorder is most consistently associated with anxious/ambivalent attachment (15, 16).

While typically emerging in childhood, SAD can persist or develop in adulthood as Adult Separation Anxiety Disorder (ASAD), with a prevalence of 6.6% and higher occurrence in women (17–19). ASAD risk factors include childhood trauma, overprotective parenting, and insecure attachment (18, 20, 21). Overprotective and intrusive parenting is thought to contribute to both childhood SAD and ASAD by limiting the development of autonomy and reinforcing dependency and fears of separation. Such parenting styles are believed to promote anxious attachment patterns and maladaptive internal working models, thereby increasing vulnerability to separation anxiety across the lifespan (17, 20, 21).

Adult separation anxiety disorder in adulthood is characterized not only by excessive distress related to separation but also by enduring behavioral and interpersonal difficulties that may impair individual functioning and close relationships (17–19). Adults with ASAD frequently exhibit heightened dependency, excessive reassurance-seeking, fear of abandonment, and limited tolerance for autonomy, which may contribute to interpersonal strain, reduced relationship satisfaction, and difficulties in emotional regulation (15, 17). In pregnant women, ASAD has been linked to heightened emotional sensitivity, increased dependence on close relationships, difficulties in affect regulation, and a higher likelihood of comorbid anxiety and mood disorders, which may also influence partner relationships and broader family dynamics (16, 22).

Pregnancy brings significant biological and psychological changes that may further heighten vulnerability to anxiety disorders, including ASAD (22). Although research specifically focusing on ASAD during pregnancy remains limited, existing evidence suggests that separation anxiety symptoms are more prevalent among pregnant women than in the general population and may intensify as pregnancy progresses (16, 22). A study conducted in Türkiye reported ASAD in 56.2% of pregnant women, underscoring the clinical relevance of separation anxiety during this period and the need for further investigation (23).

Prenatal attachment, the emotional bond between mother and fetus, is shaped by factors like gestational age, social support, and mental health (24–28). Studies consistently show that maternal depression and anxiety negatively impact prenatal attachment (29–34). A 2020 Turkish study found anxiety weakens this bond, though findings remain inconsistent (35–37). Notably, no study has yet explored the link between ASAD and prenatal attachment during pregnancy.

This study aims to assess the prevalence of ASAD in pregnant women, its relationship with attachment styles and childhood trauma, and its impact on prenatal attachment. Understanding these connections may contribute to early identification and intervention strategies, ultimately improving maternal and fetal well-being.

Guided by predefined hypotheses grounded in attachment theory and prior findings on separation anxiety and perinatal mental health, we expected adult separation anxiety disorder (ASAD) to be more prevalent among pregnant women than in the general population, given that pregnancy represents a developmental period marked by increased attachment-related concerns and heightened anxiety sensitivity (18, 19, 23, 38). We further anticipated that ASAD during pregnancy would be associated with insecure attachment patterns—particularly anxious/ambivalent attachment, which has been most consistently linked to separation anxiety—as well as with low parental care, overprotective parenting, and childhood trauma, reflecting their established roles in shaping attachment representations and separation sensitivity across development (7, 15–17, 20, 22, 39, 40). Finally, we expected ASAD during pregnancy to be negatively associated with prenatal attachment, as maternal anxiety and attachment-related difficulties have been shown to adversely affect the mother–fetus emotional bond (41–45).

## Materials and methods

The study included pregnant women in their third trimester (≥28 weeks) who sought routine obstetric care at the Göztepe Prof. Dr. Süleyman Yalçın City Hospital Gynecology and Obstetrics Clinics between October 2023 and April 2024. Of the 150 women who consented, 39 were excluded for incomplete assessments, 3 for major psychiatric disorders, and 2 for neurological diseases, resulting in 106 participants. The study was designed as a single-center, cross-sectional investigation.

The Sociodemographic Data Form, the Structured Clinical Interview for Separation Anxiety Disorder (SAD-SCI), and the Structured Clinical Interview for DSM-5 (SCID-5) were administered to the participants by a single trained psychiatrist to ensure diagnostic consistency. Participants who met at least three of the eight separation anxiety criteria, with symptoms persisting for a minimum of six months and showing clinically significant distress or impairment in functioning, were diagnosed with ASAD; based on the SAD-SCI results, participants were categorized into ASAD and control groups.

The study received ethics committee approval from İstanbul Medeniyet University Göztepe Prof. Dr. Süleyman Yalçın City Hospital Clinical Research Ethics Committee on October 11, 2023 (Decision No: 2023/0631).

### Sociodemographic data form

It includes questions about the participants’ sociodemographic characteristics such as age, marital status, education level, who they live with, income level, as well as their history, gestational week, current pregnancy process.

### Structured clinical interview for separation anxiety symptoms

The SAD-SCI, developed by Cyranowski et al. and adapted into Turkish by Diriöz et al. (2010), is a clinician-administered tool for diagnosing Adult (ASAD) and Childhood Separation Anxiety Disorder (CSAD) (38, 39). It consists of two sections with eight items each, with a diagnosis made if at least three criteria are met. The Cronbach’s alpha values are 0.56 (childhood) and 0.57 (adulthood), indicating moderate internal consistency. Test-retest reliability ranges from 0.90 to 0.92, while inter-rater reliability (Cohen’s Kappa: 0.622–0.946) is statistically significant (*p* < 0.001) (39).

### Structured clinical interview for DSM-5 disorders

SCID-5 is a clinician-administered structured interview based on DSM-5 diagnostic criteria. Developed by First et al., its Turkish adaptation, validity, and reliability were studied by Elbir et al. (40, 41). Comprising 10 modules, SCID-5 was used in this study to confirm participant diagnoses and assess psychiatric comorbidities.

### Adult separation anxiety questionnaire

Developed by Manicavasagar et al., this self-report scale assesses the severity of separation anxiety symptoms in adults (42). The Turkish validity and reliability study was conducted by Diriöz et al. (43). The 27-item scale uses a 4-point Likert format, with a cutoff score of 25 for the Turkish version. Its reliability and validity were confirmed, with a Cronbach’s alpha of 0.93 for the ASAQ (43). In the present study, Cronbach’s alpha for the ASAQ was calculated as 0.80.

### Adult attachment style scale

Originally developed by Mikulincer et al. (44), and first adapted into Turkish by Sabuncuoğlu and Berkem (45), the scale was later revised by Kesebir et al. to improve clarity. Modifications included splitting questions, changing the scoring to true/false, and expanding the scale to 18 items. It assesses secure, anxious/ambivalent, and avoidant attachment styles, with Cronbach’s alpha values of 0.72, 0.82, and 0.85, respectively (46). In the present study, Cronbach’s alpha for the AASS was calculated as 0.76.

### The Parental Bonding Inventory

Developed by Parker, Tupling, and Brown based on Bowlby’s attachment theory, this self-report scale assesses parental relationship patterns through two dimensions: care/control and overprotection (47). Adapted into Turkish by Kapcı et al. (2006), it uses a four-point Likert scale with 25 items each for mothers and fathers. High care/control scores reflect warm, accepting parental perceptions, while high overprotection scores indicate autonomy support. Cronbach’s alpha is 0.87 (mother) and 0.89 (father), with subscale consistency of 0.70 (protection) and 0.90–0.91 (care/control) (48). In the present study, Cronbach’s alpha for the PBI was calculated as 0.71.

### Childhood trauma questionnaire-33

Developed by Bernstein et al. (1994), this self-report scale assesses childhood traumatic experiences across five subdimensions: emotional neglect, physical neglect, emotional abuse, physical abuse, and sexual abuse, totaling 28 questions (49). The Turkish validity and reliability study was conducted by Şar et al. (2012) (50). and in 2021, it was revised to include an overprotection/overcontrol dimension, expanding it to the 33-item CTQ-33 (51). The scale was used to evaluate childhood trauma in pregnant participants, with Cronbach’s alpha=0.87 and a Gutmann split-half coefficient of 0.69 (51). In the present study, Cronbach’s alpha for the CTQ-33 was calculated as 0.79.

### Prenatal attachment inventory

Developed by Muller in 1993, this scale measures expectant mothers’ feelings and thoughts toward their unborn babies to assess prenatal attachment levels (52). The Turkish validity and reliability study by Yılmaz et al. confirmed its psychometric properties. This 21-item, four-point Likert-type scale assigns higher scores to stronger prenatal attachment, with a reported Cronbach’s alpha of 0.84 (53). In the present study, Cronbach’s alpha for the PAI was calculated as 0.83.

### Statistical analysis

Statistical analyses were performed using IBM SPSS Statistics version 26.0 (Statistical Package for the Social Sciences). Continuous variables with a normal distribution were presented as mean ± standard deviation, whereas non-normally distributed variables were expressed as median (interquartile range). Categorical variables were reported as frequencies and percentages.

For between-group comparisons, the independent samples t-test was used for normally distributed continuous variables, and the Mann–Whitney U test was applied for non-normally distributed variables. Effect sizes were calculated as Cohen’s d for variables analyzed using the t-test, and as the r coefficient derived by dividing the Z value by the square root of the total sample size (r = Z/√N) for variables analyzed using the Mann–Whitney U test. For Cohen’s d, values of 0.20, 0.50, and 0.80, and for r, values of 0.10, 0.30, and 0.50 were interpreted as small, medium, and large effect sizes, respectively.

The associations between adult separation anxiety symptom severity (ASAQ) and prenatal attachment (PAI) scores with other psychometric measures were evaluated using Pearson or Spearman correlation analyses, depending on data distribution. To identify factors predicting the risk of an ASAD diagnosis, multivariable logistic regression analysis was conducted, and results were reported as odds ratios (ORs) with 95% confidence intervals. Multicollinearity was assessed using tolerance and variance inflation factor (VIF) values. Model calibration was evaluated using the Hosmer–Lemeshow goodness-of-fit test, and model discrimination was assessed by receiver operating characteristic (ROC) analysis, expressed as the area under the curve (AUC).

In participants diagnosed with ASAD, hierarchical multiple linear regression analysis was performed to identify variables explaining separation anxiety symptom severity (ASAQ score). For all analyses, a two-tailed p value < 0.05 was considered statistically significant. Normality of data distribution was assessed using skewness and kurtosis values within ±1.5.

## Results

A total of 106 third-trimester pregnant women were included in the study and categorized based on the Structured Clinical Interview for Separation Anxiety Symptoms (SAD-SCI) into those with Adult Separation Anxiety Disorder (ASAD) (n=51, 48.2%) and those without ASAD (n=55, 51.8%).

The mean age was 27.78 ± 5.35 in the ASAD group and 28.36 ± 4.45 in the non-ASAD group, with no significant differences in age, education, marital status, or income level (*p>*0.05). Among women with ASAD, 98% were married, 84.3% lived with their nuclear family, 39.2% were high school graduates, and 54.9% were homemakers.

Smoking was significantly higher in the ASAD group (*p* = 0.035). Additionally, the prevalence of major depressive disorder (MDD) (*p* = 0.039), generalized anxiety disorder (GAD) (*p* = 0.001), obsessive-compulsive disorder (OCD) (*p* = 0.010), childhood separation anxiety disorder (CSAD) (*p* < 0,001) was significantly higher in the ASAD group. The Chi-Square Test also indicated a significantly higher rate of known psychiatric disorders in pregnant women with ASAD (*p* = 0.00) (**Table 1**).

**Table 1:** Comparison of the demographic and clinical features of the participants .

Parameters		Total		ASAD +		ASAD -			
		n/Mean	%/SD	n/Mean	%/SD	n/Mean	%/SD	Analysis	*p*
Age		28,08	4,89	28,36	4,45	27,78	5,35	t=0,61	0,545
Marital Status	Single	1	0,9	0	0,0	1	2,0	X^2^=1,09	0,297
	Married	105	99,1	55	100,0	50	98,0		
	Divorced	0	0,0	0	0,0	0	0,0		
Live with	Alone	4	3,8	1	1,8	3	5,9	X^2^=2,53	0,282
	Nuclear family	87	82,1	44	80,0	43	84,3		
	Extended family	15	14,2	10	18,2	5	9,8		
Education	Illiterate	0	0,0	0	0,0	0	0,0	X^2^=3,31	0,508
	Primary school	22	20,8	14	25,5	8	15,7		
	High school	41	38,7	21	38,2	20	39,2		
	College	8	7,5	3	5,5	5	9,8		
	University	31	29,2	14	25,5	17	33,3		
	Postgraduate	4	3,8	3	5,5	1	2,0		
Job	Employee	18	17,0	10	18,2	8	15,7	X^2^=3,96	0,556
	Officer	17	16,0	9	16,4	8	15,7		
	Freelance	4	3,8	3	5,5	1	2,0		
	Housewife	59	55,7	31	56,4	28	54,9		
	Student	2	1,9	0	0,0	2	3,9		
	Other	6	5,7	2	3,6	4	7,8		
Working Status	Employed	33	31,1	17	30,9	16	31,4	X^2^=0,00	0,959
	Unemployed	73	68,9	38	69,1	35	68,6		
Income level	Low	19	17,9	6	10,9	13	25,5	X^2^=3,83	0,148
	Middle	80	75,5	45	81,8	35	68,6		
	High	7	6,6	4	7,3	3	5,9		
Tobacco use	No	91	85,8	51	92,7	40	78,4	**X^2^=4,45**	**0,035**
	Yes	15	14,2	4	7,3	11	21,6		
Alcohol use	No	106	100,0	55	100,0	51	100,0	-	-
	Yes	0	0,0	0	0,0	0	0,0		
History of medical illness	No	89	84,0	49	89,1	40	78,4	X^2^=2,23	0,135
	Yes	17	16,0	6	10,9	11	21,6		
Regularly used medication	No	90	84,9	48	87,3	42	82,4	X^2^=0,50	0,480
	Yes	16	15,1	7	12,7	9	17,6		
Comorbid mental illness	No	73	68,9	48	87,3	25	49,0	**X^2^=0,00**	**0,000**
	Yes	33	31,1	7	12,7	26	51,0		

SD, Standard Deviation; t, Independent Samples t-test; X^2^, Chi-Square Test; ASAD, Adult Separation Anxiety Disorder.

Bold values indicate statistical significance.

The mean Adult Separation Anxiety Questionnaire (ASAQ) score was significantly higher in the ASAD group (30.98 ± 12.79) than in the non-ASAD group (12.64 ± 8.14) (*p* < 0.001).

Significantly higher mean scores in the Parental Bonding Inventory (PBI) subscales for pregnant women without ASAD, and higher scores in other listed measures for those with ASAD, are detailed in **Table 2**.

**Table 2:** Comparison of psychometric measurements between pregnant women with and without adult separation anxiety disorder.

Parameters	ASAD -		ASAD +				
	Mean	SD	Mean	SD	Analysis	p	Effect size
ASAQ	12,64	8,14	30,98	12,79	**t=-8,873**	**<0,001**	**1,73**
PAI	62,76	10,60	64,55	10,97	t=-0,852	0,396	0,17
PBI- Maternal Care	42,16	9,12	37,25	11,63	**t=2,427**	**0,017**	**0,47**
PBI- Maternal Overprotection	13,53	3,96	10,73	4,53	**t=3,399**	**0,001**	**0,66**
PBI- Mother Total	55,82	10,09	47,98	12,14	**t=3,624**	**<0,001**	**0,71**
PBI- Paternal Care	38,44	12,58	34,38	13,16	t=1,610	0,110	0,32
PBI- Paternal Overprotection	13,81	4,74	12,74	4,58	t=1,173	0,243	0,23
PBI- Father Total	52,26	12,17	47,12	13,69	**t=2,026**	**0,045**	**0,40**
AASS- Secure Attachment	3,56	1,48	3,18	1,56	t=1,314	0,192	0,25
AASS- Avoidant Attachment	2,51	1,39	3,08	1,48	**t=-2,044**	**0,043**	**0,40**
AASS- Anxious/Ambivalent Attachment	1,20	1,03	2,16	1,64	**t=-3,627**	**<0,001**	**0,71**
CTQ-33- Physical abuse	5,55	2,27	6,14	2,80	**Z=-3,165**	**0,002**	**0,31**
CTQ-33- Emotional abuse	5,85	2,24	7,34	3,00	**Z=-4,473**	**<0,001**	**0,43**
CTQ-33- Sexual abuse	5,07	,54	6,26	3,22	**Z=-3,223**	**0,001**	**0,31**
CTQ-33- Physical neglect	7,47	3,51	8,48	3,70	t=-1,431	0,155	0,28
CTQ-33- Emotional neglect	9,02	3,87	10,96	5,06	**t=-2,222**	**0,028**	**0,43**
CTQ-33- Overprotection	8,51	3,58	10,08	4,42	**t=-2,009**	**0,047**	**0,39**
CTQ-33- Total	41,65	11,42	49,06	15,24	**t=-2,833**	**0,006**	**0,55**

SD, Standart Deviation; t, Independent samples t-test; Z, Mann Whitney U test; ASAD, Adult Separation Anxiety Disorder; ASAQ, Adult Separation Anxiety Questionnaire; PAI, Prenatal Attachment Inventory; PBI, The Parental Bonding Inventory; AASS, Adult Attachment Style Scale; CTQ-33, Childhood Trauma Questionnaire-33. Effect sizes were calculated as Cohen’s d for comparisons performed using the independent samples t-test, and as r (Z/√N) for comparisons conducted using the Mann–Whitney U test.

Bold values indicate statistical significance.

Pearson Correlation Analysis revealed a significant negative correlation between ASAQ scores and PBI subscales, including maternal care/control (*p* = 0.002), maternal overprotection (p<0.001), maternal total (p<0.001), paternal care/control (*p* = 0.017), paternal overprotection (*p* = 0.024), and paternal total (*p* = 0.001).

A significant positive correlation was found between ASAQ scores and AASS subscales for avoidant (*p* < 0.001) and anxious/ambivalent attachment (*p* < 0.001), as well as CTQ-33 subscales for physical neglect (*p* = 0.030), overprotection (*p* = 0.002), total CTQ-33 score (*p* < 0.001), and Spearman analysis confirmed correlations with physical abuse (*p* = 0.023), emotional abuse (*p* < 0.001), and sexual abuse (*p* < 0.001).

When effect sizes were examined (**Table 2**), large effects were observed for ASAQ (d = 1.73), PBI–Mother Total (d = 0.71), PBI–Maternal Overprotection (d = 0.66), AASS–Anxious/Ambivalent Attachment (d = 0.71), and CTQ–Emotional Abuse (r = 0.43). Moderate effect sizes were identified for PBI–Maternal Care (d = 0.47), PBI–Father Total (d = 0.40), AASS–Avoidant Attachment (d = 0.40), CTQ–Physical Abuse (r = 0.31), CTQ–Sexual Abuse (r = 0.31), CTQ–Emotional Neglect (d = 0.43), and CTQ–Total (d = 0.55). Small effect sizes were observed for PAI (d = 0.17), PBI–Paternal Care (d = 0.32), PBI–Paternal Overprotection (d = 0.23), AASS–Secure Attachment (d = 0.25), and CTQ–Physical Neglect (d = 0.28).

Pearson analysis showed a positive correlation between PAI scores and PBI subscales for paternal care/control (p=0.004) and total paternal involvement (*p* = 0.034), but a negative correlation with paternal overprotection (*p* = 0.050), while Spearman analysis identified a negative correlation with CTQ-33 sexual abuse (*p* = 0.012) (**Table 3**).

**Table 3:** The relationship between adult separation anxiety questionnaire and prenatal attachment inventory scores with other psychometric measurement scores.

Parameters		ASAQ	PAI
ASAQ	r	-	
	p	-	
PAI	r	0,101	
	p	0,302	
PBI- Maternal Care	r	**-0,301**	0,072
	p	**0,002**	0,461
PBI- Maternal Overprotection	r	**-0,355**	-0,068
	p	**<0,001**	0,489
PBI- Mother Total	r	**-0,405**	0,042
	p	**<0,001**	0,670
PBI- Paternal Care	r	**-0,234**	**0,280**
	p	**0,017**	**0,004**
PBI- Paternal Overprotection	r	**-0,221**	**-0,193**
	p	**0,024**	**0,050**
PBI- Father Total	r	**-0,310**	**0,208**
	p	**0,001**	**0,034**
AASS- Secure Attachment	r	-0,090	0,081
	p	0,357	0,406
AASS- Avoidant Attachment	r	**0,335**	0,044
	p	**<0,001**	0,654
AASS- Anxious/ Ambivalent Attachment	r	**0,516**	0,058
	p	**<0,001**	0,557
CTQ-33- Physical Abuse	rho	**0,221**	0,053
	p	**0,023**	0,594
CTQ-33- Emotional Abuse	rho	**0,381**	-0,109
	p	**<0,001**	0,270
CTQ-33- Sexual Abuse	rho	**0,359**	**-0,246**
	p	**<0,001**	**0,012**
CTQ-33- Physical Neglect	r	**0,212**	-0,144
	p	**0,030**	0,143
CTQ-33- Emotional Neglect	r	0,172	-0,072
	p	0,079	0,467
CTQ-33- Overprotection	r	**0,306**	-0,187
	p	**0,002**	0,056
CTQ-33- Total	r	**0,358**	-0,187
	p	**<0,001**	0,057

r, Pearson correlation analyses; rho=Spearman correlation analyses ASAQ, Adult Separation Anxiety Questionnaire; PAI, Prenatal Attachment Inventory; PBI, The Parental Bonding Inventory; AASS, Adult Attachment Style Scale; CTQ-33, Childhood Trauma Questionnaire-33.

Bold values indicate statistical significance.

The multivariable logistic regression model was statistically significant (model χ² = 38.929; *p* = 0.001). The explanatory power of the model, as indicated by Nagelkerke R², was 0.420, with an overall classification accuracy of 71.8%. Model discrimination, evaluated using receiver operating characteristic (ROC) analysis, yielded an area under the curve (AUC) of 0.819 (95% CI: 0.740–0.898; *p* < 0.001), while model calibration was deemed adequate based on the Hosmer–Lemeshow goodness-of-fit test (χ² = 10.86; *p* = 0.210) (**Table 4**).

**Table 4:** The effectiveness of PAI, PBI, AASS, and CTQ-33 scores in increasing the risk of adult separation anxiety disorder diagnosis.

Parameters	B	S.E.	Wald	df	p	Odds ratio	95% Cl	
							LB	UB
PAI	0,023	0,027	0,708	1	0,400	1,023	0,970	1,080
PBI- Mother Total	-0,072	0,038	3,605	1	0,058	0,930	0,864	1,002
PBI- Father Total	0,008	0,032	0,064	1	0,800	1,008	0,947	1,073
AASS- Secure Attachment	-0,124	0,201	0,377	1	0,539	0,884	0,595	1,312
AASS- Avoidant Attachment	-0,144	0,216	0,447	1	0,504	0,866	0,567	1,321
AASS- Anxious/ Ambivalent Attachment	0,593	0,261	5,155	1	0,023	1,810	1,085	3,021
CTQ-33-Physical Abuse	0,076	0,109	0,482	1	0,487	1,079	0,871	1,335
CTQ-33- EmotionalAbuse	0,062	0,136	0,204	1	0,652	1,063	0,814	1,389
CTQ-33- Sexual Abuse	0,741	0,380	3,807	1	0,051	2,098	0,997	4,417
CTQ-33- Physical Neglect	-0,073	0,085	0,741	1	0,389	0,930	0,788	1,097
CTQ-33- Emotional Neglect	-0,030	0,087	0,121	1	0,727	0,970	0,818	1,151
CTQ-33- Overprotection	-0,167	0,101	2,725	1	0,099	0,846	0,694	1,032

SE, Standard Error; Cl, Confidence Interval; LB, Lower Bound; UB, Upper Bound; NR2=0,38, X2=34,58, p<0,001, PAI, Prenatal Attachment Inventory; PBI, The Parental Bonding Inventory; AASS, Adult Attachment Style Scale; CTQ-33, Childhood Trauma Questionnaire-33. The model was co-adjusted for age, current obsessive–compulsive disorder, and the CTQ-33 minimization subscale. No multicollinearity was detected (all tolerance values > 0.20 and variance inflation factor [VIF] < 5). Model calibration was assessed using the Hosmer–Lemeshow goodness-of-fit test and was found to be adequate (χ² = 10.860; p = 0.210). Model discrimination was supported by a Nagelkerke R² of 0.420 and an overall classification accuracy of 71.8% (model χ² = 38.929; p = 0.001). Discriminative performance was further evaluated using receiver operating characteristic (ROC) analysis, yielding an area under the curve (AUC) of 0.819 (95% CI: 0.740–0.898; p < 0.001).

After co-adjustment for age, current obsessive–compulsive disorder, and the CTQ-33 minimization subscale, a statistically significant association was observed between a diagnosis of adult separation anxiety disorder (ASAD) and anxious/ambivalent attachment style (OR = 1.810; 95% CI: 1.085–3.021; *p* = 0.023). Regression analysis showed that childhood sexual abuse was associated with an approximately twofold increased likelihood of an ASAD diagnosis; however, this association remained at a borderline level of statistical significance (*p* = 0.051). Prenatal attachment was not associated with ASAD diagnosis (OR = 1.023; 95% CI: 0.970–1.080), with a small effect size and confidence intervals excluding a clinically meaningful increase in risk (**Table 4**). The lack of association between prenatal attachment and ASAD diagnosis remained unchanged after adjustment for parental bonding variables and childhood trauma domains, including sexual abuse.

According to the Hierarchical Linear Regression Analysis, among ASAD cases, only AASS-Anxious/Ambivalent scores were found to be a significant predictor of ASAQ scores (*p* = 0.004) (**Table 5**).

**Table 5:** The effectiveness of PAI, PBI, AASS, and CTQ-33 scores in explaining adult separation anxiety questionnaire scores in cases diagnosed with adult separation anxiety disorder.

Model		Unstandardized coefficients		Standardized coefficients	t	p	95% Cl	
		B	SE	Beta			LB	UB
1	(Constant)	44,341	10,702		4,143	,000	22,812	65,870
	PAI	-,198	,163	-,174	-1,214	,231	-,526	,130
2	(Constant)	52,282	11,728		4,458	,000	28,660	75,904
	PAI	-,101	,174	-,089	-,583	,563	-,452	,249
	PBI-Mother Total	-,165	,172	-,159	-,955	,345	-,512	,183
	PBI-Father Total	-,137	,167	-,145	-,820	,417	-,474	,200
3	(Constant)	24,958	14,130		1,766	,085	-3,557	53,474
	PAI	-,058	,159	-,051	-,364	,717	-,379	,263
	PBI-Mother Total	,014	,167	,014	,085	,933	-,324	,352
	PBI-Father Total	-,194	,159	-,205	-1,225	,228	-,514	,126
	AASS-Secure Attachment	1,845	1,302	,231	1,417	,164	-,783	4,473
	AASS- Avoidant Attachment	1,684	1,367	,202	1,232	,225	-1,075	4,442
	**AASS- Anxious/ Ambivalent Attachment**	**3,410**	**1,098**	**,445**	**3,105**	**,003**	**1,194**	**5,625**
4	(Constant)	-3,397	24,520		-,139	,890	-52,916	46,122
	PAI	,016	,166	,014	,097	,923	-,319	,351
	PBI-Mother Total	,208	,215	,200	,964	,340	-,227	,642
	PBI-Father Total	-,182	,157	-,192	-1,160	,253	-,499	,135
	AASS-Secure Attachment	2,141	1,304	,268	1,642	,108	-,492	4,775
	AASS- Avoidant Attachment	2,324	1,426	,279	1,630	,111	-,555	5,204
	**AASS- Anxious/ Ambivalent Attachment**	**3,276**	**1,090**	**,428**	**3,006**	**,004**	**1,075**	**5,476**
	CTQ-33 Total	,228	,162	,279	1,407	,167	-,099	,554

SE, Standard Error; Cl, Confidence Interval; LB, Lower Bound; UB, Upper Bound; PAI, Prenatal Attachment Inventory; PBI, The Parental Bonding Inventory; AASS, Adult Attachment Style Scale; CTQ-33, Childhood Trauma Questionnaire-33 For Model 1, the coefficient of determination was R² = 0.03, with an adjusted R² of 0.001 (F = 1.47, p = 0.231). For Model 2, R² was 0.09 and adjusted R² was 0.03 (F = 1.54, p = 0.216). Model 3 demonstrated an R² of 0.30 and an adjusted R² of 0.20 (F = 3.01, p = 0.015), while Model 4 showed an R² of 0.33 with an adjusted R² of 0.22 (F = 2.93, p = 0.014). No multicollinearity was detected, as all tolerance values.

Bold values indicate statistical significance.

## Discussion

Our study examined the relationship between adult separation anxiety disorder (ASAD) in pregnant women and sociodemographic factors, pregnancy-related factors, attachment styles, childhood traumas, and prenatal attachment by comparing those with and without ASAD. To our knowledge, this is the first study to investigate the link between ASAD and prenatal attachment during pregnancy, as well as the first in Turkiye to explore ASAD in relation to attachment styles and childhood trauma in pregnant women.

The mean age of pregnant women was 27.78 ± 5.35 in the ASAD group and 28.36 ± 4.45 in the non-ASAD group, with no significant difference between groups, consistent with previous studies (22, 54). While most studies found no link between maternal age and ASAD, one study reported higher ASAD rates in younger mothers (23). In our study, 98% of the ASAD group and 100% of the non-ASAD group were married, with no significant differences in marital status, education, employment, occupation, or income. These findings align with previous research suggesting that sociodemographic factors do not play a major role in ASAD during pregnancy (22, 23, 54). This highlights the need to focus on psychological, emotional, and attachment-related factors in ASAD development.

Our study evaluated comorbid psychiatric disorders in groups with and without separation anxiety disorder, revealing that pregnant women with ASAD had higher rates of major depressive disorder, generalized anxiety disorder (GAD), and obsessive-compulsive disorder (OCD) compared to those without ASAD. The literature indicates a strong comorbidity between separation anxiety disorder and other psychiatric conditions. Studies report that anxiety disorders are five times more common and mood disorders four times more common in individuals with separation anxiety disorder than those without it. Among ASAD patients, psychiatric comorbidity rates average 91%, with major depressive disorder at 42.5%, GAD at 14%, and OCD at 20% (19, 55). These findings align with existing research, emphasizing the need for further investigation into psychiatric comorbidities in individuals with ASAD.

The prevalence of ASAD among third-trimester pregnant women in our study was 48.2%. Although research on separation anxiety during pregnancy is limited, previous studies report similar rates. The literature indicates a lifetime prevalence of 4.8%, with 43.1% of cases developing after age 18 (18). ASAD appears to be more common during pregnancy than in the general population, with an estimated prevalence of 45% (16, 54), increasing further in the third trimester (22, 23). Our findings align with existing research.

Physiological changes during pregnancy, along with concerns related to miscarriage and childcare, have been shown to be associated with increased general anxiety symptoms (56, 57). In addition, pregnancy-specific stressors, such as fears regarding the child’s well-being and the approaching delivery, have been reported to exacerbate perinatal distress (58). During this period, perinatal anxiety is often characterized by heightened worries about fetal safety and potential harm, which have been linked to more pronounced separation anxiety symptoms during pregnancy (59) In this context, the higher prevalence of ASAD observed in the third trimester compared with the general adult population may reflect a transient increase in separation-related distress associated with the developmental characteristics of pregnancy rather than a distinct clinical phenomenon. Consistent with this interpretation, longitudinal studies indicate that adult separation anxiety symptoms emerging during pregnancy frequently diminish in the postpartum period (16, 60), suggesting that pregnancy-related ASAD may represent a temporary intensification of attachment-related anxiety rather than a persistent adult-onset disorder.

In our study, cigarette use was significantly higher in the ASAD group than in the non-ASAD group. This finding is consistent with limited research indicating that smokers, including students, exhibit higher levels of separation anxiety than non-smokers (61). Additionally, a study on anxiety and depression risk factors during pregnancy reported significantly higher anxiety levels among pregnant smokers (62). Higher rates of smoking among women with ASAD may be understood in the context of increased psychiatric comorbidity, as ASAD has been frequently associated with other anxiety and mood disorders, which are themselves linked to higher smoking prevalence (19, 63). Taken together, these findings suggest that smoking among pregnant women with ASAD may be related to underlying anxiety burden and comorbid psychiatric symptoms, highlighting the need for integrated assessment and support during pregnancy.

The relationship between ASAD and attachment styles showed that both anxious/ambivalent and avoidant attachment were significantly associated with greater separation anxiety severity in pregnant women. Anxious/ambivalent attachment was more prevalent in the ASAD group and increased the risk of ASAD diagnosis by 1.8 times, also significantly explaining ASAD severity. These findings align with previous studies. Research on pregnant and postpartum women has linked separation anxiety disorder to anxious attachment (16). Similarly, a 2009 study on panic disorder patients found an association between separation anxiety and anxious/ambivalent attachment, with a weaker link to avoidant attachment (15). A 2023 study in Turkiye also identified anxious/ambivalent attachment as a risk factor for separation anxiety (14). Our findings support existing literature, suggesting that attachment disruptions may contribute to the severity of ASAD.

In this study, parenting attitudes reflecting pregnant women’s retrospective perceptions of how they were parented by their own caregivers during childhood showed that caring, autonomy-supportive, and tolerant parenting styles were inversely associated with ASAD severity, whereas overprotective parenting behaviors were associated with increased ASAD severity. Pregnant women diagnosed with ASAD reported more negative parental bonds compared with those without ASAD, particularly lower perceived maternal care and higher levels of overprotection. These findings are consistent with previous studies linking overprotective or intrusive parenting styles to separation anxiety disorder and other anxiety disorders (17, 20, 21, 64).

Early life experiences and parenting attitudes, particularly those related to maternal behavior, play a critical role in the development of psychopathology, including ASAD. Considering that insecure mother–child attachment and negative parenting attitudes increase the risk of mental disorders, it is important to address recurrent interpersonal conflicts reported by pregnant women in their relationships with their own mothers. Supporting secure attachment, promoting autonomy-supportive parenting approaches, and strengthening positive intergenerational parenting practices may contribute to the prevention of ASAD.

We found a significant positive correlation between childhood trauma severity and ASAD symptoms. Regression analysis showed that childhood sexual abuse was associated with an approximately twofold increased likelihood of an ASAD diagnosis; however, this association remained at a borderline level of statistical significance. Early adverse life events and childhood trauma are well-established risk factors for ASAD (17, 65, 66), with studies showing that individuals with ASAD report more childhood traumatic experiences than those without (67, 68). A study in Turkiye also found a positive correlation between childhood trauma, particularly emotional and sexual abuse, and adult separation anxiety severity (69). Additionally, the literature supports that trauma, especially sexual abuse, is a predictor of anxiety disorders during pregnancy (63, 70). These findings suggest that childhood trauma may be associated with weakened coping mechanisms, as well as heightened sensitivity to separation and difficulties in emotion regulation, and that these processes may contribute to increased vulnerability to ASAD.

Our study found no significant relationship between ASAD and prenatal attachment levels. While some studies report an inverse correlation between anxiety and prenatal attachment (33, 34, 36, 71), others found no significant link (35, 37) A meta-analysis suggested that anxiety has a small effect on prenatal attachment, which becomes more apparent with larger sample sizes and higher study quality (72). To our knowledge, this is the first study examining the relationship between ASAD and prenatal attachment. The absence of a significant association may be attributed to sample size or measurement tools, underscoring the need for further research.

Our study found an inverse correlation between childhood sexual abuse and prenatal attachment severity, suggesting that pregnant women with a history of sexual trauma had lower levels of prenatal attachment. The literature on this relationship is limited, but a 2023 study supports our findings, showing that childhood traumas negatively impact prenatal attachment (73), Another study similarly reported that a history of interpersonal trauma adversely affects prenatal attachment (74). These findings highlight the significant influence of early life trauma on the mother-infant bond, offering valuable insights into this underexplored area.

In our study, we examined how the expectant mother’s bond with her parents and upbringing memories influenced prenatal attachment. Prenatal attachment was positively correlated with the father’s caring parenting attitudes and the strength of the father-child bond. While most research has focused on the mother-daughter relationship, highlighting the role of a secure maternal bond in enhancing prenatal attachment (75–78), studies on paternal influence remain limited. Our findings align with previous research showing a positive correlation between paternal care and prenatal attachment (74). This highlights the significance of a supportive father-child relationship in fostering a strong maternal-infant bond, offering a new perspective on the role of paternal influence in prenatal attachment.

Our study also found that retrospectively perceived overprotective paternal parenting attitudes were associated with higher levels of prenatal attachment. This finding is consistent with the limited existing literature examining paternal parenting experiences and prenatal attachment, with one international study reporting a positive association between perceived paternal overprotectiveness and prenatal attachment (79). Another study observed a similar association during the first trimester, although this effect appeared to diminish in later stages of pregnancy (80). These findings suggest that, in some women, particularly those reporting limited or inconsistent early interactions with their fathers, paternal overprotectiveness may be perceived as a form of care, which could contribute to stronger prenatal attachment.

## Limitations

The inclusion of only third-trimester pregnant women limited the evaluation of separation anxiety symptoms in earlier trimesters, preventing the assessment of ASAD onset timing. The cross-sectional design restricted the ability to establish causal relationships between variables. Additionally, the reliance on self-report scales for attachment styles and childhood traumas may have introduced reporting bias, potentially influencing result interpretation.

## Conclusion

Pregnancy is a vulnerable period for the development of psychopathology, during which adult separation anxiety disorder (ASAD) appears to be particularly common. Considering the role of insecure attachment patterns and adverse parenting experiences in the development of ASAD and other psychiatric disorders, promoting secure attachment is critical for fostering healthy generations. From a clinical perspective, these findings support the potential value of implementing routine screening for adult separation anxiety disorder during late pregnancy, along with brief, targeted interventions for women with insecure attachment patterns or histories of childhood trauma. Future longitudinal studies should examine the longer-term clinical and developmental consequences of separation anxiety emerging during pregnancy by investigating the postpartum course of ASAD and its effects on parenting behaviors, mother–infant relationships, and child developmental outcomes using standardized assessment measures.

## Data Availability

The raw data supporting the conclusions of this article will be made available by the authors, without undue reservation.
